# The Hippo signaling pathway modulates pancreatic tissue homeostasis

**DOI:** 10.1038/s41420-025-02636-0

**Published:** 2025-07-24

**Authors:** Xiaoyan Wang, Jiajing Du, Haiyun Li, Zhiwei Cao, Zhonghua Cheng, Zuoyun Wang

**Affiliations:** 1 Department of Anatomy and Histoembryology, Shanghai Pudong Hospital, Fudan University, Shanghai, China; 2 Department of Gastroenterology, Huadong Hospital; Department of Geriatrics, Shanghai Key Laboratory of Clinical Geriatric Medicine, Huadong Hospital, Fudan University, Shanghai, China; 3 Department of Gastroenterology, Shanghai Xuhui Center Hospital, Shanghai, China

**Keywords:** Cell biology, Cell growth

## Abstract

The Hippo signaling pathway, a highly conserved signaling cascade from *Drosophila* to mammals, plays a critical role in mammals in regulating cell proliferation, senescence and apoptosis. In the pancreas, this pathway has emerged as a key regulator in various processes, including development, cell fate determination, and regeneration following injury. Consequently, aberrant the Hippo signaling pathway disrupts pancreatic tissue homeostasis, leading to uncontrolled cell proliferation, oncogenic transformation, and the subsequent development of pancreatic diseases. In this review, we comprehensively summarize the multifaceted roles of the Hippo signaling pathway in both physiological and pathological aspects of the pancreas, such as pancreas development, pancreatitis, pancreatic ductal adenocarcinoma and diabetes. Furthermore, we highlight the potential mechanisms and new therapies targeting the Hippo signaling pathway in pancreatic diseases, which helps to address unresolved issues in research.

## Facts

- Inhibition of YAP/TAZ promotes endocrine cell differentiation, whereas activation maintains progenitor cell proliferation (e.g., MPCs) or drives acinar-to-ductal metaplasia.
- YAP activation is a key driver of PDAC initiation and progression, promoting tumor cell proliferation, metabolic reprogramming, and the formation of an immunosuppressive microenvironment by regulating downstream targets such as STAT3, JAK-STAT, and MYC.
- In both acute and chronic pancreatitis, aberrant activation of YAP/TAZ upregulates factors such as CTGF and SPP1, leading to the activation of pancreatic stellate cells (PSCs), fibrosis, and inflammatory cascades.
- MST1 activation promotes β-cell apoptosis via the AKT-JNK-BIM pathway, whereas YAP overexpression protects β-cells and enhances their proliferation, suggesting a dynamic balance in β-cell homeostasis regulated by the Hippo pathway.
- Loss of LATS1/2 or MST1/2 results in aberrant YAP/TAZ activation, leading to pancreatic developmental defects (such as acinar dedifferentiation into ductal-like structures) or disease progression, including PDAC and diabetes.

## Open questions

- How does the Hippo pathway coordinate with Wnt, TGF-β, and other signaling pathways to regulate pancreatic development and disease?
- How can YAP/TAZ activity be precisely controlled during pancreatic regeneration to prevent tumorigenesis (e.g., PDAC)?
- How do YAP/TAZ coordinate interactions between pancreatic cancer cells and stromal cells (e.g., CAFs, immune cells)?
- Can targeting Hippo signaling in the tumor microenvironment improve the immune response in PDAC therapy?
- How do small-molecule inhibitors of MST1 or LATS2 (e.g., IHMT-MST1-39) balance β-cell protection with systemic side effects (such as EGFR inhibition)?

## Introduction

The pancreas is a vital digestive organ with both exocrine and endocrine functions. The exocrine pancreas, constituting approximately 95% of the organ’s total parenchymal volume, is principally organized into two functionally integrated components: clustered acinar cells responsible for biosynthesis of hydrolytic enzymes (including lipolytic, proteolytic, and amylolytic agents) and an intricate branching duct network that conveys these zymogen-rich secretions toward the duodenal lumen. The pancreatic endocrine compartment governs systemic glucose regulation through a sophisticated islet microarchitecture, wherein five distinct endocrine cell populations synthesize phylogenetically conserved peptide hormones. These specialized islet constituents include: (i) glucagon with hyperglycemic effects (derived from α-cells), (ii) anabolic, storage-promoting polypeptides (specific to β-cells), (iii) paracrine inhibitory regulators (secreted by δ-cells), (iv) appetite-modulating enteropancreatic peptides (produced by PP-cells), and (v) orexigenic gastric mediators (restricted to ε-cells). This cellular consortium functions through dynamic intercellular crosstalk to maintain metabolic homeostasis. These cells, which differentiate from the same progenitors, cluster in small groups called islets of Langerhans [1, 2].

Pancreatic homeostasis is closely related to the onset of various diseases, including pancreatitis, pancreatic cancer and diabetes. Acute pancreatitis is often triggered by excessive alcohol consumption, gallstones, and genetic susceptibility, accompanied by abnormal secretion of digestive enzymes such as lipases and amylases [3]. Persistent inflammatory responses in the exocrine pancreas can trigger aberrant acinar-to-ductal metaplasia (ADM), wherein acinar cells dedifferentiate into ductal-like cells. This pathological process can ultimately lead to chronic pancreatitis (CP), characterized by fibrosis. Notably, patients with CP face a significantly elevated risk of developing pancreatic cancer, approximately 10 to 15 times greater than that of the general population. Pancreatic ductal adenocarcinoma (PDAC) composes approximately 90% of all pancreatic malignancies, solidifying its position as the predominant subtype. Islet cell tumors represent the second most common form, typically presenting at a younger age than PDAC. Other less common pancreatic neoplasms include cystic lesions, mucinous cystadenomas, epithelial neoplasms, and pancreatoblastomas [4]. Correspondingly, endocrine islet dysfunction is a direct etiological factor in the development of diabetes mellitus, which is primarily categorized into type 1 and type 2. Type 1 diabetes mellitus fundamentally results from autoimmune-mediated β-cell destruction leading to absolute insulin deficiency, whereas type 2 diabetes mellitus primarily arises from insulin resistance in peripheral target tissues (particularly skeletal muscle, liver, and adipose tissue) combined with relative insulin deficiency through progressive β-cell dysfunction [5].

**Fig. 1:**
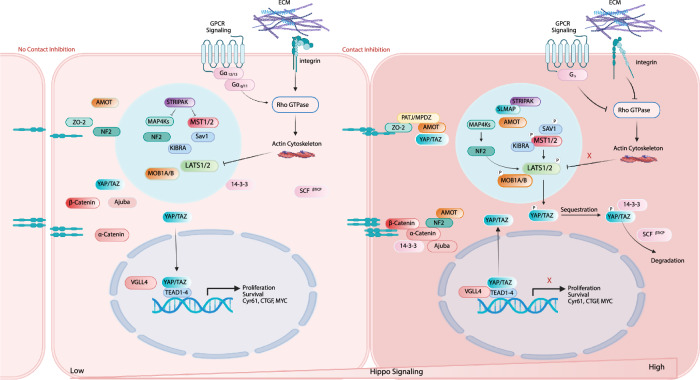
Hippo signaling pathway regulation schematic. Cell junctions, extracellular matrix (ECM) properties, and G protein-coupled receptor (GPCR) signaling are among the many factors that regulate the phosphorylation cascade of the core Hippo signaling pathway kinases (MST1/2–LATS1/2), thereby influencing the localization and activity of YAP/TAZ and controlling the expression of their downstream target genes. When the Hippo signaling pathway is highly active, YAP/TAZ are phosphorylated, leading to their retention in the cytoplasm or degradation, which suppresses their transcriptional activity. In contrast, when the activity of the Hippo signaling pathway kinases is low, YAP/TAZ translocate into the nucleus, where they bind to TEAD1–4 and promote the expression of genes involved in cell proliferation and survival.

Hippo signaling pathway is an evolutionarily conserved pathway that exhibits a core function of regulating organ size and maintain tissue homeostasis through the precise modulation of cell fate decisions [4]. When the Hippo signaling pathway is on, MST1/2 interact with the scaffold protein SAV1 and phosphorylate it [5, 6]. SAV1 fosters MST1/2 to phosphorylate LATS1/2 kinases. LATS1/2 binds to MOB1, which also facilitates the phosphorylation of LATS1/2 [7]. The activated LATS1/2-MOB1 complex subsequently phosphorylates the downstream transcriptional coactivators YAP and its paralog TAZ. Once phosphorylated, YAP/TAZ associate with 14-3-3 proteins, leading to their retention in the cytoplasm [8]. SCF^β-TrCP^E3 ligase also interacts with the phosphorylated YAP/TAZ, regulating the ubiquitination and proteasome-dependent degradation [9, 10] (Fig. 1). On the contrary, when the Hippo signaling pathway is off, YAP/TAZ enters the nucleus and acts as the co-activator of downstream transcription factors. TEAD family transcription factors (TEADs), with four homologous proteins, are the most common and conserved downstream mediators. TEADs binds to nuclear YAP/TAZ, involving in the binding of target gene promoters for cell proliferation, growth, and survival. Vestigial like family member 4 (VGLL4) competitively binds to YAP/TAZ with TEAD, inhibiting downstream target gene expression [11].

The Hippo signaling pathway, in collaboration with TGF-β, Wnt, and other signaling pathways, coordinates the regulation of pancreas development, regeneration, and the onset of associated diseases [12–14]. Abnormalities in the Hippo signaling pathway affect pancreatic physiology in specific ways. For instance, pancreas-specific deficiency or overexpression of the core Hippo kinases, MST1/2 or YAP, leads to abnormal pancreas development [15]. Concerning pancreatic cancer, YAP/TAZ has been reported as a master driver of PDAC with a poor prognosis [16]. In this review, we mainly focus on the regulation of Hippo signaling pathway in pancreas development and pancreatic diseases, such as pancreatitis pancreatic cancers and diabetes.

## Pancreas cell lineage determination and morphogenesis

### Timeline and regulators of mouse early pancreas development

Pancreatic organogenesis, which is highly similar in humans and mice, can be divided into the following two stages characterized by distinct morphological features. Diverse signals from neighboring tissues and transcription factors in different cell lineages work together to determine the cell fate.

#### The embryonic specification phase (E8.5-12.5)

**Fig. 2:**
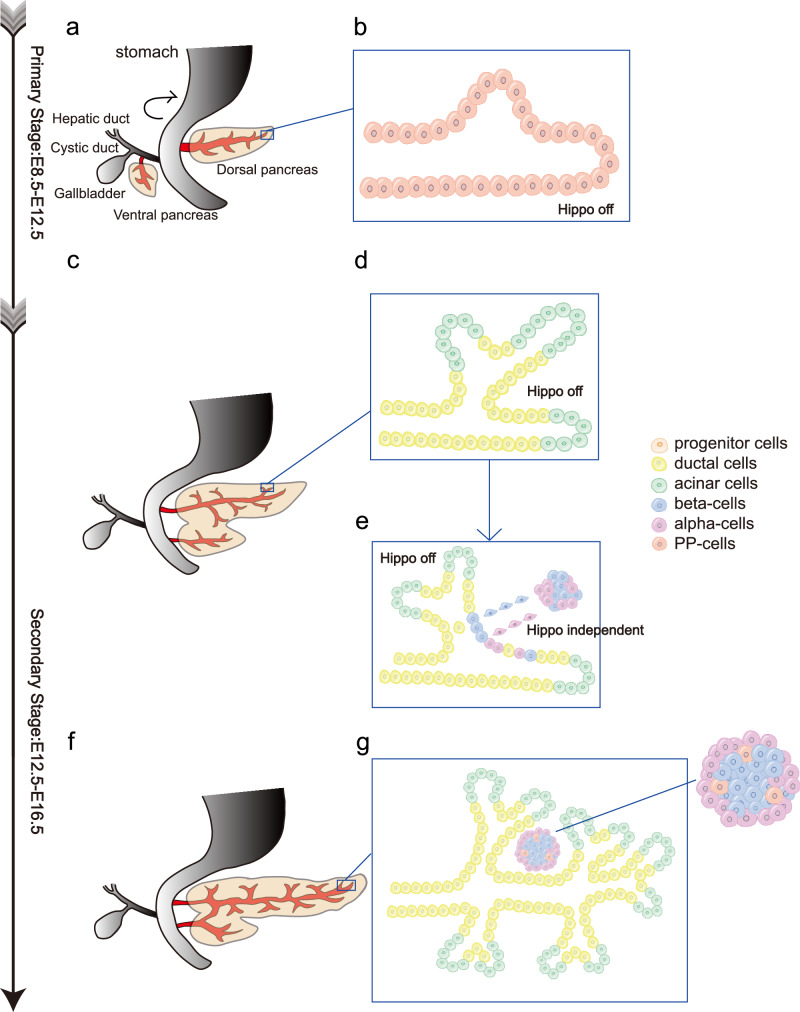
Pancreatic organogenesis in mice and the role of Hippo pathway in cell lineage determination. Ventral and dorsal pancreatic buds form a single organ by E12.5. Following extensive branching, cellular differentiation, and migration, the pancreatic ductal epithelium gives rise to exocrine cells (green) and endocrine cells (blue and pink). The endocrine cells subsequently migrate into the surrounding mesenchyme, where they contribute to the formation of pancreatic islets. **a** Emergence of independent dorsal and ventral pancreatic primordia from the foregut endoderm at around E9.5. **b** During the primary stage of development, Hippo pathway suppression enables rapid proliferation of undifferentiated pancreatic progenitor cells, supporting the growth of dorsal and ventral buds. **c** Integration of dorsal and ventral buds into a single organ by E12.5 after elongation and gut rotation. **d, e** The onset of the secondary transition at E12.5 marks the compartmentalization of the pancreatic epithelium into trunk (duct/endocrine precursors) and tip (acinar precursors) domains, with the trunk containing bi-potent cells that initially differentiate into β- and α-cells between E13.0 and E15.0. **f, g** During late embryogenesis (E16.5–18.5), differentiated cells migrate and organize to form the mature pancreas, with PP cells emerging and endocrine cells moving from the center to the periphery near exocrine tissues.

The primary stage patterns the initiation of pancreatic organogenesis and the proliferation of pancreatic progenitor cells. Upon completion of endoderm specification at E7.5, the independent dorsal and ventral pancreatic primordia emerge on opposing lateral aspects of the foregut endoderm, approximating E9.5 [17] (Fig. 2a). Following elongation and gut rotation, the two pancreatic buds integrate to form a single organ by E12.5 (Fig. 2c). In this stage, the pancreatic progenitor cells, which are still undifferentiated, proliferate at a rapid speed to support the growth of dorsal and ventral pancreatic buds (Fig. 2b).

**Fig. 3:**
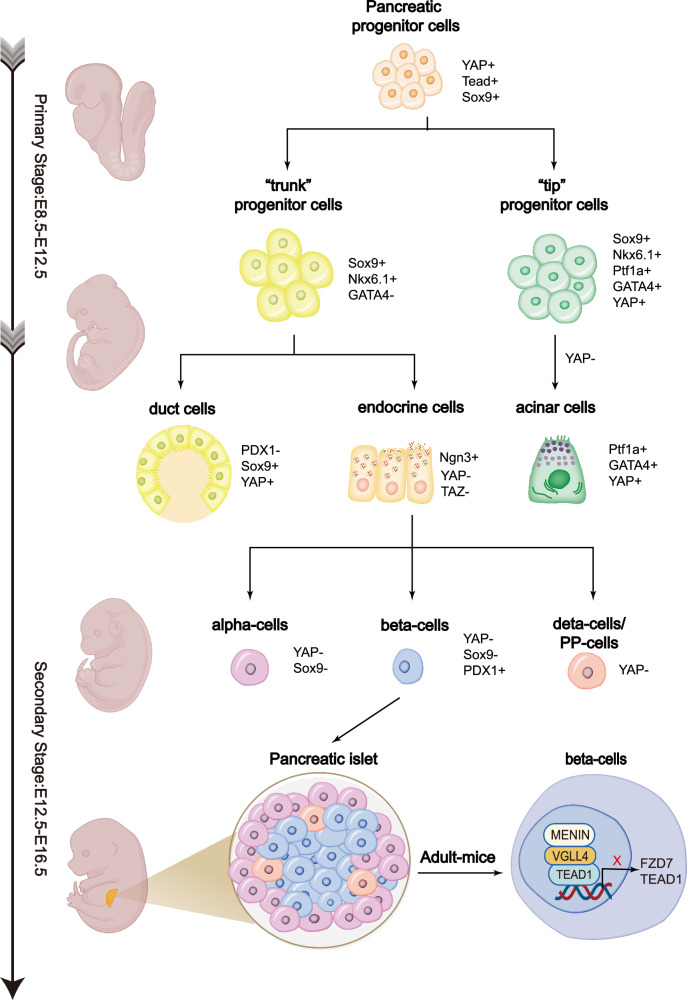
The expression of Hippo kinases and relevant genes during the three stages of normal pancreas development. Nuclear YAP accumulation is observed mainly in pancreatic progenitor cells and exocrine regions (duct cells and acinar cells), but is almost undetectable in differentiated endocrine cells. See text for details.

Although in this stage progenitor cells are undifferentiated, there exist specific gene expressions that determine the direction of future cell differentiation and compartmentalization. Pancreas duodenum homeobox-1(PDX-1), is the “master regulator” of various differentiation factors during the pancreatic embryonic development. PDX-1 marks the pre-pancreatic endoderm before E8.5-E9.0 [18], playing influential roles in regulating the elongation and fusion of dorsal and ventral buds into a single organ. PDX-1 is eventually restricted to β-cells and δ-cells in islets, thus regulating the expression of relevant hormones [19] (Fig. 3). Ptf1a, which co-expressed with PDX-1, also determines the pancreatic fate and mediates the expansion of multipotent pancreatic cells (MPCs). As the development proceeds, Ptf1a directs the specification of acinar cells and is eventually restricted to acinar cells [20] (Fig. 3). Additionally, Sox9, which finally remains in ductal cells, plays a similar role in early pancreas development as Ptf1a [21]. Other transcription factors, such as Hb9, Sox17, GATA4/6 and Hnf family, participate in the specification and proliferation of progenitor cells in the primary stage [18, 22–24].

#### The morphological construction phase (E12.5 to postnatal)

The secondary stage is characterized by cell lineage allocation and differentiation. The pancreatic progenitor cells begin to differentiate and the pancreatic epithelium compartmentalizes into central duct-like structures (“trunks”) and more peripheral clustered cells (“tips”) at around E12.5 [2] (Fig. 2d). The tip domain comprises multipotent pancreatic cells (MPCs) that differentiate into acinar cells by E13.5, contributing to the exocrine functions of the pancreas. The trunk domain consists of bi-potent cells that later differentiate into whether ductal structures or endocrine cells. Among the five types of endocrine cells, β-cells and α-cells emerge first (at E13.0–15.0), followed by δ-cells [25].

The compartmentalization and differentiation during the secondary stage also require specific transcription factors. Cells in both the trunk domain and the tip domain are Sox9 + /NKX6.1 + . However, cells in the trunk domain contain little GATA4, while the tip domain is characterized by GATA4 expression, which can still be detected in acinar cells after differentiation [2]. Sox9 is important for expressing the bHLH transcription factor NEUROG3 (Ngn3), which is responsible for differentiating trunk cells into endocrine cells [26]. Post-Ngn3 differentiation, Sox9 is absent from endocrine cells but present in duct cells [27].

During E16.5 to the postnatal stage, the already differentiated cells further migrate to form the mature morphology of pancreas (Fig. 2f). At E18.0–18.5, following the emergence of PP-cells, endocrine cells initially develop in the central region of the pancreas before migrating toward the periphery, where they aggregate near the exocrine structures [28] (Fig. 2g). Various signaling factors mediate the process, but further molecular mechanisms remain to be discovered.

### Hippo-specific roles for cell fate decisions

#### The Hippo signaling pathway regulates MPCs via the YAP/TEAD/SOX9 axis

Cebola et al. utilized human embryonic stem cells (hESCs) to differentiate into stage-specific pancreatic progenitor cells and discovered that nuclear YAP interacts with TEAD transcription factors. Moreover, YAP is significantly enriched in MPCs. The YAP-TEAD complex then facilitates the transcription of some MPC enhancers, including Sox9, thus driving MPCs expansion and growth of pancreas epithelium [29] (Fig. 3). The report of YAP co-expressing with MPCs markers Sox9 and Ptf1a offers further support for the conclusion that the Hippo signaling pathway governs MPCs partially through the YAP/TEAD/SOX9 axis [30].

#### The Hippo signaling pathway controls “trunk” and “tip” cell differentiation

A portion of cells in the “trunk” region are strongly YAP positive and subsequently differentiate into duct cells, which display the highest YAP expression eventually. The rest “trunk” cells are YAP null and subsequently differentiate into YAP null endocrine cells [15, 29]. The “trunk” cells induced by Ngn3 mainly differentiate into endocrine cells through hippo-independent and hippo-dependent pathways, with the former being the main pathway. YAP inhibition can promote Ngn3+ endocrine precursors to differentiate into β-cells and deplete YAP-positive ductal-like progenitor cells, while YAP expression restricts the endocrine differentiation [31] (Fig. 3). George et al. reported the overlap of positive Ngn3 and negative YAP in the same cells [15], which also supports the conclusion.

Additionally, deletion of upstream kinases in the Hippo signaling pathway can enhance YAP expression and induce ectopic endocrine differentiation. Wu et al. demonstrated that specific knockout of LATS1/2 in endocrine progenitor cells promoted ductal expansion and halting endocrine differentiation [32], as the deletion of LATS1/2 fostered YAP/TAZ expression and turned on KRT19 expression. They indicated that the inhibition of YAP/TAZ mediated by hippo upstream kinases in endocrine progenitors is a precondition for endocrine regulation and differentiation [32]. In endocrine cells, YAP is absent at birth but restores its expression in two weeks postnatally [33]. Collectively, these mechanisms suggest that the YAP-null environment induced by Ngn3 and Hippo upstream kinase is required in endocrine differentiation.

The “tip” cells mainly differentiated into acinar cells, and this process is Hippo-dependent. YAP expression, which is induced by the knockout of Hippo upstream regulators, leads to ectopic acinar structures [15, 33] (Fig. 3), indicating that YAP repression is necessary for acinar cell differentiation. As mentioned above, LATS1/2KO also halts the acinar differentiation except for endocrine differentiation and leads to immune infiltration [32]. George et al. delineated that YAP expression is robust in the nuclei of cells within the “tip” region, and upon their differentiation into acinar cells, YAP translocates to the cytoplasm while maintaining high expression levels [15]. As multiple studies have shown a close relationship between yap and proliferation, different YAP locations of duct and endocrine cells cause one-third of duct cells and acinar cells (YAP + ) to be still proliferative, while only 5% of endocrine cells (YAP-) remain active in the cell cycle after birth. In essence, the Hippo signaling pathway becomes functionally active when repressing YAP expression in acinar differentiation.

#### Regulation of embryonic development and cellular proliferation by the Hippo Signaling Pathway

As both acinar and endocrine differentiation requires a YAP repression, lack of upstream Hippo kinases results in YAP activation and thus irregular development. Both George et al. and Gao et al. deleted MST1/2 (MST1/2KO) in pancreatic epithelium within early development and discovered similar abnormal phenotypes, including decreased pancreas mass, increased cell proliferation, disorganized structures of islets, immune infiltration and de-differentiation of acinar cells to ductal-like structures [15, 33]. Differently, George et al. reported that ratios of β-cells to α-cells remained constant [14], while Gao et al. displayed an increasing α/β ratio [33]. These two studies explain the contradiction of decreased pancreas mass and increased cell proliferation in different ways. George et al. proposed that the MST1/2KO-induced lack of a continuous ductal system caused the release of digestive enzymes and subsequent auto-digestion. Therefore, the necrosis counteracts the proliferation promoted by the MST1/2KO/β-catenin/c-myc axis [15]. Gao et al. attributed it to the postnatal alternation in acinar phenotype. They suggested that the aberrant pancreatic mass and structure were dependent on the postnatal alternations. Absence of MST1/2 had no impact on embryonic YAP expression, yet it triggered an increased YAP expression level after postnatal day 7 (P7), demonstrating that MST1/2 depletion does not interfere with acinar cell differentiation. However, the MST1/2KO pancreas is unable to retain the acinar identity. Consistent with YAP expression, the acinar-specific MST1/2KO model showed that the absence of MST1/2 promoted ductal metaplasia after P7. The trans-differentiation then triggered cell death, which helps explain the paradox between upregulated proliferation and reduced pancreatic mass [33]. Pancreatic endocrine specification and differentiation are critically regulated by the Hippo signaling pathway through suppression of YAP1/TAZ in endocrine progenitors, thereby ensuring proper islet architecture and restricting ectopic endocrine lineage commitment [32].

With respect to LATS1/2, Braitsh et al. developed the LATS1/2KO model in pancreas and reported similar abnormalities with Wu et al. in development. Braitsh et al. identified a novel mechanism where LATS1/2 knockout leads YAP/TAZ to activate VNN1, resulting in cysteamine production that stimulates both ROS and NF-κB signaling the activated NF-κB then initiated aberrant EMT, which impeded the epithelial morphogenesis and the secondary developmental stage [34]. However, LATS1/2 deletion in postnatal pancreas displays a different phenotype. Liu et al. demonstrated the formation mechanism of the pancreatitis-like phenotype with the Ptf1a^CreER^ system that deletes LATS1/2 in mature acinar cells [35]. The absence of LATS1/2 stimulates CTGF and SPP1 via YAP/TAZ, which precedes PSCs activation. PSCs activation recruits immune cells and further causes pancreatic inflammation and fibrosis. These two studies reveal that YAP/TAZ is the downstream effector of LATS1/2 that directly regulates pancreas development. Hence, LATS1/2 suppression of YAP/TAZ activity plays a crucial role in pancreas development. In addition, the latest research identifies MENIN as TEAD1 corepressors in β cells. Li et al. show thatVGLL4 and MENIN bound to TEAD1 to disrupting Wnt signaling pathway, and leading to impaired β cell proliferation [36]. The synthesis of these studies reveals that the Hippo signaling pathway is instrumental in governing cell proliferation and upholding pancreatic endocrine homeostasis.

## The Hippo signaling pathway in pancreatitis: from pathogenesis to regeneration

### Hippo-dependent pathogenic mechanisms and hazard factors in pancreatitis

Pancreatitis can be divided into acute pancreatitis (AP) and chronic pancreatitis (CP). AP features damage to the acinar cells, which may be caused by gallstone-induced pancreatic duct obstruction, ethanol, hyperlipemia, and abnormal release of calcium [37]. The acinar damage further leads to the activation of trypsinogen to trypsin within these cells. Subsequently, immune cells will be recruited for immune response, which precedes acinar cell death via apoptosis or oncosis. Recent evidence suggests that AP, especially the recurrence of AP, can lead to duct damage and fibrosis, eventually progressing to CP [38, 39]. Besides, alcohol and tobacco abuse, diabetes, and obesity may also contribute to CP [40, 41]. CP is characterized by immune cell infiltration, ADM, chronic inflammation, and fibrogenesis. Activated PSCs are responsible for fibrosis in CP, while the inflammation is mainly attributed to macrophages [40, 42]. Although the morphological features of CP are clear, the precise pathogenesis of CP has not been delineated yet. Fortunately, several studies have revealed the molecular mechanisms involved in AP and CP, and some of them correlate with Hippo/YAP signaling.

#### Acute pancreatitis

In murine models, AP is often induced by short-term cerulein administration. Liu et al. demonstrated that after cerulein treatment, the expression of YAP/TAZ in acinar cells increased significantly, while LATS1 decreased and TEAD1 remained constant [35]. Similarly, pancreatitis-induced ADM cooperates with oncogenic KRAS to drive pancreatic cancer progression by activating YAP1/TAZ, which in turn upregulate JAK-STAT3 signaling, promoting PDAC development [43]. However, Liu et al. suggested that YAP upregulation could be caused by Hippo pathway disruption (LATS1/2 deletion) [35]. Gu et al. linked the activation of YAP with the activity of RNAs, finding that miR194, which was responsible for inhibiting YAP, was downregulated in cerulein-induced AP. MALAT1, a lncRNA that competes with YAP for miR194, is upregulated in AP further to restrain the expression of YAP [44].

As a key mediator of AP, the activation of YAP in acinar cells drives the progression of the disease by triggering the expression of downstream genes. For example, YAP upregulates CTGF and SPP1, which activate PSCs and contribute to fibrosis development [35]. During AP progression, the activation of PSCs occurs prior to immune cell infiltration, setting the stage for ADM and acinar cell death [35].

#### Chronic pancreatitis

As CP is mostly distinguished by PSC-induced fibrosis, studies of CP mainly focus on how Hippo signaling pathway regulates the activity of PSCs. Recent studies utilize various approaches to induce CP, including repeated and prolonged exposure to cerulein, pancreatic ductal ligation, or tail vein injection of dibutyltin dichloride (DBTC) [44, 45]. In a murine CP model, Tamura et al. discovered a decreased expression of PTEN (a PI3K pathway component) and SAV1 (an upstream Hippo kinase) in acinar cells, which was caused by the downregulation of CCAAT/enhancer-binding protein-α (CEBPA) [44]. They further extended the downstream regulating mechanism that simultaneous inhibition of PTEN and SAV1 induced higher levels of p-AKT and nuclear YAP, which facilitated the activation of surrounding PSCs and macrophages by increasing CTGF expression [44]. In line with this finding, the role of YAP in PSCs activation has also been verified in DBTC-induced CP where knockdown of YAP in PSCs interfered with DNA replication and cell proliferation [45]. In addition, the activity of YAP in PSCs can be stimulated by TGF-β1, but repressed by IFN-γ.

### Pancreatitis reparation and exocrine pancreas regeneration via self-replication or the “redifferentiation”

As damage in AP is mainly concentrated in acinar cells, the regeneration of exocrine cells is crucial for the recovery of AP. Acinar cell regeneration can be achieved via self-replication or the “redifferentiation” from duct-like acinar cells back to acinar cells [46]. Although YAP was once reported to promote acinar cell regeneration in the pancreatitis models, these regeneration models have rarely been reported to have a connection with Hippo signaling pathway, but are more closely related to Hedgehog, Notch, and Wnt pathways instead.

Additionally, although pancreatic progenitor cells are rare in the exocrine pancreas, they can also be regenerated by exocrine cells. Using lineage tracing experiments and time course analysis, Panciera et al. substantiated that specific expression of YAP^S127A^ in acinar cells converted these cells into ductal pancreatic progenitors, which could be passaged in the absence of YAP/TAZ subsequently [47].

Concisely, lineage-specific YAP overexpression appears to play a critical role in the regeneration of both endocrine and exocrine pancreas. The understanding then provokes our further thoughts on whether the inhibition of upstream Hippo kinases (MST1/2, LATS1/2) could activate YAP expression and facilitate the pancreas regeneration process. However, it has only been substantiated in other organ systems, such as hepatocytes and pituitary endocrine lineages [48, 49], and diabetes therapies. Therefore, more studies in the normal pancreatic regeneration model are expected to be carried out to test the hypothesis.

## Hippo signaling pathway orchestrates the multistep progression of PDAC

The pancreas cancer is a highly malignant cancer with strong invasiveness. Exocrine tumors are relatively much more common, in which the PDAC subtype accounts for the majority. PDAC evolves from normal ductal epithelium or acinar cells to precursor lesion through to malignancy and metastasis [50]. PDAC frequently metastasizes to liver or lymph nodes [51], and is often associated with poor prognosis. Patients diagnosed with PDAC exhibit a 5-year survival rate of only about 11% [52]. The poor prognosis of PDAC is attributed to poor methods of early detection and effective therapeutic. Most patients with PDAC often remain asymptomatic in the early stage, and there are no effective therapeutics for PDAC treatment, partly because of the drug resistance [53]. We will focus on the molecular pathogenic mechanism and relevant therapies of PDAC in this review.

### Deregulation of hippo signaling pathway in the occurrence and development

PDAC can arise from acinar cells or ductal cells (centroacinar and duct cells) in situ [54]. In ductal cells, the mutation of oncogenes (KRAS, TP53) will directly cause invasive PDAC [55, 56]. However, acinar cells undergo a series of transitions to initiate PDAC. Under specific conditions (such as pancreatitis), acinar cells first transdifferentiate into ductal-like cells through the ADM process, which is a crucial step in the initiation of PDAC. ADM then triggers pancreatic intraepithelial neoplasia (PanIN) that precedes invasive PDAC [57, 58]. Besides, acinar cells can also be induced to form intraductal papillary mucinous neoplasms (IPMN) under specific conditions, and progress to PDAC under sustained pressure such as tumor suppressor gene mutations. Krushna C. Patra et al. found that the simultaneous occurrence of GNAS and KRAS mutations in acinar cells can form low-level IPMN by constructing KGC^ER^ mice (Ptf1a^CreER^; KRAS^G12D^; Rosa26-LSL-rtTA; TetO-Gnas^R201C^). If p53 mutations occur simultaneously (KGPC^ER^ mice, Ptf1a^CreER^; KRAS^G12D^; Tp53 LoxP/+; Rosa26-LSL-rtTA; TetO-Gnas^R201C^), it can further develop into PDAC on the basis of IPMN [59]. Therefore, both acinar and ductal cells can progress to PDAC due to oncogenic mutations. However, acinar and ductal cells have different susceptibility to oncogenes, different tendencies towards forming precancerous lesions, and significant differences in subtypes and prognosis of tumor progression. In other words, the intrinsic characteristics of the originating cell can greatly affect the fate of tumor cells.

The tumor microenvironment is essential to the development of PDACs. It is characterized by cancer cells and the surrounding dense stroma which contains CAFs, MDSCs, immune cells, immune-suppressive leukocytes, endothelial cells and extracellular matrix [60–63]. Various external signals can activate the PSCs, the major component of CAFs, by upregulating YAP and TAZ expression in these cells, and the proliferation of PSCs promotes tumor growth [64, 65]. In addition, many signaling pathways regulate the localization of YAP in cells by affecting LATS1/2, the upstream activator of YAP, thus affecting the occurrence and development of PDAC. Insulin/IGF-1 receptor and GPCR systems engage in crosstalk, promoting YAP activation through the PI3K and PKD pathways. Additionally, YAP is a primary mediator of pro-oncogenic mutant p53, with p53 concurrently exerting negative regulation on YAP via PTPN14 activation [66] (Fig. 3).

**Fig. 4:**
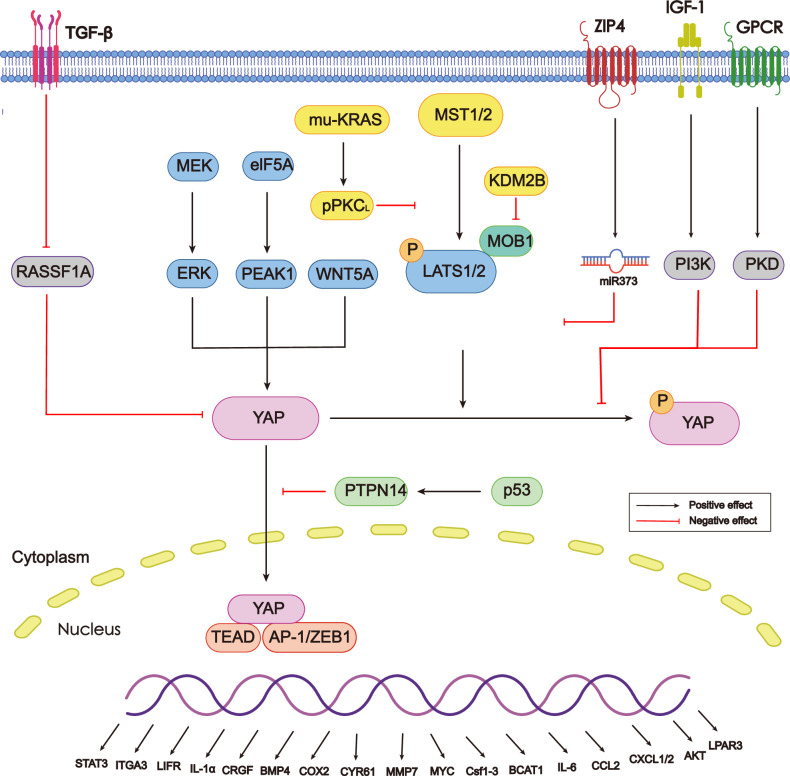
A schematic review of key genes that regulate PDAC initiation, progression, metastasis, and recurrence. YAP/TEADs complex plays a pivotal role in PDAC via transcription of downstream effectors that directly induce cell transformation and proliferation (STAT3, LIFR, BMP4, CTGF, COX2, MMP7, BCAT1, CYR61 and MYC), promote EMT (ITGA3, AKT, LPAR3) or maintain a stable tumor microenvironment (IL-6, Csf1-3, CCL2, CSCL1/2). Distinct upstream regulators including MAPK signaling pathway and WNT5A regulate the nuclear translocation of YAP in either Hippo-dependent or Hippo-independent pathways.

Oncogenic KRAS mutation, which has been reported as the leading hallmark of PDAC, plays a crucial role in both the initiation and maintenance of PDAC [67]. Additionally, as the tumor progresses, one KRAS mutant allele often leads to genomic loss of the remaining KRAS allele [68]. Downstream of KRAS, YAP acts as a vital transcriptional switch to control the transcription of genes involved in neoplastic proliferation, progression toward migration, invasion and immune surveillance (Fig. 4). Complementally, those factors upstream of KRAS mutation, such as obesity, altered gut microbiota and inflammation, can amplify the downstream YAP signaling via receptors on the cell membrane (e.g., RTK, GPCR), thus inducing more rapid PDAC progression [69] (Fig. 4).

Hence, the Hippo signaling pathway, especially YAP signaling, closely correlates with the development process of PDAC. We will then review the role of Hippo signaling in the order of different stages of the development of PDAC, encompassing initiation, progression, metastasis and recurrence.

#### PDAC initiation: ADM to early-stage PanIN

ADM is the earliest pre-neoplastic lesion in PDACs derived from acinar cells, making it the most essential symbol of PDAC initiation. Utilizing genetically engineered mouse models (GEMMs), recent studies have uncovered the important role of YAP/TAZ in the induction of ADM. The initiation of PDAC is generally attributed to either pancreatitis or oncogenic KRAS mutation (mu-KRAS). Gruber et al. generated a caerulein-induced pancreatitis model in R26-LSL-YFP; LSL-KRAS^G12D^; Ela1-Cre^ERT2^ mice to demonstrate that YAP and TAZ in acinar cells were required to induce ADM and PanIN formation in the context of pancreatitis [43]. They delineated the mechanism that YAP/TAZ-induced upregulation of STAT3 and LIFR (JAK–STAT3 pathway components) through TEAD4 increased the sensitivity to inflammatory cytokines, which further led to the trans-differentiation of acinar cells and ADM lesions. Consistently, Wang et al. reported that YAP was indispensable for PDAC initiation in mu-KRAS models. KRAS^G12C^ activated pPKCι signaling that quenched LATS1 phosphorylation, resulting in YAP nuclear activation and neoplastic transformation of pancreatic cells [70] (Fig. 4). Contrary to the above findings, Zhang et al. proposed that YAP was dispensable for ADM induction in oncogenic KRAS and pancreatitis models [71]. Nimmakayala et al. found that YAP cooperates with PAF1 during ADM and development, and verteporfin and CA3 inhibit ADM by targeting the PAF1/YAP/SOX9 axis [72]. Thus, future studies are required to confirm the function of YAP/TAZ in acinar cell-derived PDAC initiation and resolve this contradiction. In addition, some non-Hippo pathway proteins may regulate the occurrence of ADM by mediating YAP expression. Loss of FBXW7 leads to the accumulation of its substrates, including YAP and c-Myc, which causes ADM and PanIN formation that precede PDAC initiation [73].

Studies on the initiation of PDACs that derive from ductal cells are rare. Park et al. discovered that in KRAS-mutant mice, the pancreatic duct cell-specific knockout of LATS1 and LATS2 led to the rapid emergence of carcinomatous features in situ, triggered by the co-activation of YAP and AP-1 [74] (Fig. 4). Thus, it can be confirmed that YAP/AP-1 complex acts as the downstream mediator of mut-KRAS in duct cell-derived PDAC initiation.

Essentially, YAP plays a pivotal role in PDAC initiation by inducing ADM (in acinar cells) or carcinoma in situ (in ductal cells). However, few studies focus on the function of upstream Hippo kinases, and thus it remains unclear whether Hippo kinases upstream of YAP regulate YAP activity in PDAC initiation.

#### PDAC progression and maintenance

In most reported cases of PDAC, which originate from acinar cells, ADM and early-stage PanIN progress to late-stage PanIN and eventually develop into PDAC. This progression and subsequent maintenance of PDAC phenotype require the participation of Hippo pathway kinases, especially YAP. The expression of several Hippo kinases upstream of YAP, such as MST1, MOB1 and NF2 [75, 76], have been reported to be reduced in PDAC cancer tissues, which indicates that these kinases may function in PDAC progression and maintenance. Low expression of MST1 in PDAC inhibits the activity of caspase-1, which is responsible for cancer cell pyroptosis, and thus promotes PDAC development [77] (Fig. 4). Quan et al. demonstrated that MOB1 suppression achieved by KDM2B decreased levels of pLATS and pYAP/TAZ, which facilitated YAP/TAZ nuclear translocation and PDAC cell proliferation [75] (Fig. 4). Taken together, these findings suggest that MST1 regulates PDAC development in a Hippo-independent manner, while decreased expression of NF2 and MOB1 depends on Hippo pathway to facilitate PDAC progression and maintenance.

While upstream Hippo kinases play minor roles in PDAC development, YAP serves as the central mediator of Hippo signaling, driving the progression and maintenance of PDAC. YAP is mediated by various upstream axes in different subtypes of PDACs, regardless of whether or not they depend on KRAS mutation. For instance, syndecan binding protein (SDCBP) [78] SGLT2-induced hnRNPK nuclear translocation [79], WDR3-induced GATA4 nuclear localization [80], eIF5A-PEAK1 signaling [81] and MEK-ERK (MAPK signaling pathway) [71] all directly interact with YAP to promote its transcription and expression in the nuclei of PDAC cells (Fig. 4). Moreover, the crosstalk between the Hippo signaling pathway and other signaling pathways, such as the regulation of YAP activity by TGF-β [82], also plays a crucial role in the maintenance of PDAC. WNT5A, which is enriched in PDAC, suppresses the phosphorylation of LATS1/2 and YAP, thereby facilitating YAP nuclear translocation and promoting tumor growth [83]. In parallel, TGF-β enhances the nuclear accumulation of the YAP/SMAD2 complex by downregulating RASSF1A, a key scaffold protein in the Hippo pathway (Fig. 4).

YAP/TEAD effectors regulate genes driving PDAC proliferation, metabolism, immunity, and tumor microenvironment. YAP/TEADs directly sustain the proliferation of KRAS-mutant cancer cells by promoting the transcription of target genes, including BMP4, CTGF, COX2, MMP7, BCAT1, CYR61 and MYC [68, 71]. Moreover, Murakami et al. discovered the metabolic and immune function of YAP target genes, which provide proper conditions for PDAC cell proliferation. YAP cooperates with TEADs to transcript genes encoding multiple metabolic enzymes by directly enhancing the expression of MYC, which maintains metabolic homeostasis, especially nucleotide synthesis, that is required for PCs proliferation and survival [84]. UDP-glucose pyrophosphorylase 2 (UGP2), the direct targets of YAP/TEADs complexes, regulates glycogen synthesis and protein N-glycosylation, whose deletion halters tumor growth [85]. Under hypoxia, Unc-51 like kinase 1 and 2 (ULK1/2) kinase upregulates pyruvate kinase M2 (PKM2) and facilitates glycolysis, which may be mediated by ULK1/2-induced phosphorylation of YAP at Ser227 [86]. To maintain an immune suppressive environment for PDAC development, YAP is activated in PCs to drive the transcription of IL-6 and Csf1-3 which are responsible for the polarization of MDSCs in bone marrow [87]. YAP/TEADs complex also elevates the expression of CXCL1/2 and CCL2 to recruit the differentiated MDSCs into the pancreas, which further inhibits the influx of MHCII^+^ cells and T cells. Therefore, the apoptosis of neoplastic ductal cells will be restrained, which is conducive to PDAC maintenance.

Genes downstream of YAP also regulate tumor microenvironment, which is important to PDAC development (Fig. 4). Zhang et al. unraveled that loss of YAP in neoplastic epithelial cells decreased CAFs, collagen, and immune infiltrates, which are the main components of the stroma. The finding suggests that besides the function of stimulating cell proliferation, YAP-target genes (COX2, MMP7, IL-1α, and IL-6) are also responsible for stromal response [71]. YAP is also closely relevant to PSC activities. It can be detected in both pancreatic cancer cells (PCs) and PSCs, another key member of the stroma [86]. In PCs, YAP regulates the interaction of PSCs and PCs via the production and release of CTGF, which is a paracrine cytokine that activates PSCs [88]. Once PSCs are activated by these paracrine cytokines from PCs or direct contact with PCs, YAP will be upregulated in these cells to suppress the expression of MMP3, a protease that degrades ECM proteins. Hence, the ECM property can be maintained, which is conducive to PCs proliferation. Additionally, activation of PSCs correlates with decreased apoptosis of PCs and adds to the viability of PDAC cells as well [88]. Soft stroma promotes YAP1 degradation through the autophagic-lysosomal pathway, suppressing malignancy [89].

#### PDAC metastasis

PDAC metastasis is characterized by cell detachment, migration and invasion. EMT is regarded as a vital mediator of cell migration. The Hippo signaling pathway has been reported to be involved in the process. PRMT5-mediated methylation of MST2 inhibits the Hippo signaling pathway, promoting PDAC progression and metastasis, while PRMT5 inhibition restores Hippo pathway activity and suppresses tumor growth [90]. Likewise, NF2 silence also facilitates PDAC metastasis [91]. Loss of these upstream Hippo kinases gives rise to the activation of YAP/TAZ [92], which directly regulates EMT and metastasis. Furthermore, Hippo signaling pathway also mediates PDAC metastasis by regulating the pentose phosphate pathway (PPP). The R5P and NADPH supplied by PPP are crucial for the synthesis of nucleotides, fatty acids, sterols, and the clearance of reactive oxygen species. Previous studies have shown that metabolic reprogramming of PPP leads to malignant progression of PDAC and resistance to KRAS^G12D^ inhibitor [93]. The prolactin receptor-short isoform (PRLR-SF) is involved in activating Hippo signaling pathway in PDAC cells by directly interacting to NEK9, decreasing YAP nuclear localization and its binding to TEAD1, downregulating G6PD/TKT, and inhibiting PPP [94]. However, the acidic microenvironment formed by tumor cells promotes the binding of YAP to TEAD4, enhances the expression of the target gene MPP1, and thus leads to the invasion and migration of PDAC [95]. In general, YAP induces EMT in PCs via hyperactivation of its target genes or signals, such as pAKT signaling and ITGA3 [92, 96].

Studies on the upstream regulators of YAP in PDAC metastasis, especially those related to Hippo kinases, have been rare. It has only been discovered that ZIP4 could inhibit LATS2 via the activation of miR373, which then promoted the nuclear translocation of YAP and its combination with ZEB1 [96]. YAP/ZEB1 complex cooperated with TEADs to transcript ITGA3, which contributed to EMT plasticity and cell adhesion. To conclude, YAP is of central importance in the regulation of EMT and PDAC metastasis, which provides new ideas for the treatment of metastatic pancreatic cancer.

#### Hippo-YAP Mediates PDAC Recurrence

Recent studies suggest that PDAC recurrence is largely dependent on the augmentation of YAP signaling. Kapoor et al. used a doxycycline-inducible KRAS^G12D^ transgene GEMM to clarify the role of YAP in PDAC recurrence. Although the extinction of the oncogenic KRAS mutation, which was achieved by doxy withdrawal, was efficient in the regression of PDAC, 70% of the mice still developed relapsed tumors [97]. KRAS mutation was re-observed in half of these cases, while the remaining relapsed tumors featured KRAS^G12D^-independent YAP amplification [97]. They also demonstrated the mechanism of YAP substituting for mut-KRAS in tumor maintenance: YAP/TEAD2 complex activated genes that governed cell cycle and DNA replication via cooperation with E2F transcription factors, which fostered the growth of relapsed tumors (Fig. 4). However, the specific mechanism of the initiation of PDAC recurrence is still unclear. Future researches are expected to work out which upstream regulators promote the amplification of YAP in relapsed PDAC and whether upstream Hippo kinases function during the process.

### Therapeutic strategies targeting the Hippo signaling pathway in PDAC

As indicated above, YAP plays a pivotal role in the initiation, development, metastasis and recurrence of both KRAS-mutant PDACs and PDACs independent of KRAS mutation [83]. Consequently, it is plausible to develop drugs or explore relevant genes that mainly target YAP in PDAC treatment.

Drugs inhibiting the activity of YAP in different ways have been proven efficient in PDAC treatment. Verteporfin, which has been used clinically in the treatment of various cancer types, can also inhibit PDAC cell proliferation and survival by abrogating the interaction between YAP and TEAD [98]. VGLL4 was discovered as a competitor of YAP in combination with TEADs. Therefore, VGLL4-mimicking peptides hold promise as therapeutic agents for cancers driven by YAP-mediated cell proliferation [99]. Additionally, tyrosine kinase inhibitors such as dasatinib and pazopanib enhance YAP phosphorylation and block its nuclear translocation [100], potentially suppressing PDAC progression. However, the function of VGLL4 and these tyrosine kinase inhibitors has not yet been verified in PDAC, which deserves further studies.

Furthermore, TEAD inhibition combined with broad-spectrum RAS-GTP inhibition has been identified to be a promising candidate combination therapeutic regimen to overcome monotherapy resistance [101]. Therefore, a dual inhibition therapy targeting both YAP/TEAD and YAP/AP-1 binding should be developed in response to this subtype of PDAC. Drugs disrupting the interaction of YAP and AP-1 are expected to be explored in future research.

## The Hippo signaling pathway and diabetes mellitus

### The Hippo signaling pathway and its impact on β-cell fate in diabetes mellitus

Diabetes mellitus features the loss of mass or function of β-cells responsible for producing insulin. Loss of β-cells is a hallmark shared by both type 1 and type 2 diabetes mellitus (T1DM and T2DM) [102, 103]. Among various types of cell death (necrosis, apoptosis, autophagy), apoptosis is the major form in β-cells. However, the mechanism of β-cell apoptosis differs subtly between T1DM and T2DM. In T1DM, autoimmune reactions trigger the secretion of cytokines and the activation of inflammatory cells, which precede the apoptosis of β-cells [104]. In contrast, in T2DM, metabolic stress induces the secretion of pro-inflammatory cytokines, which causes peripheral insulin resistance. This, in turn, leads to a high demand for insulin and β-cells, ultimately driving β-cell apoptosis [105].

**Fig. 5:**
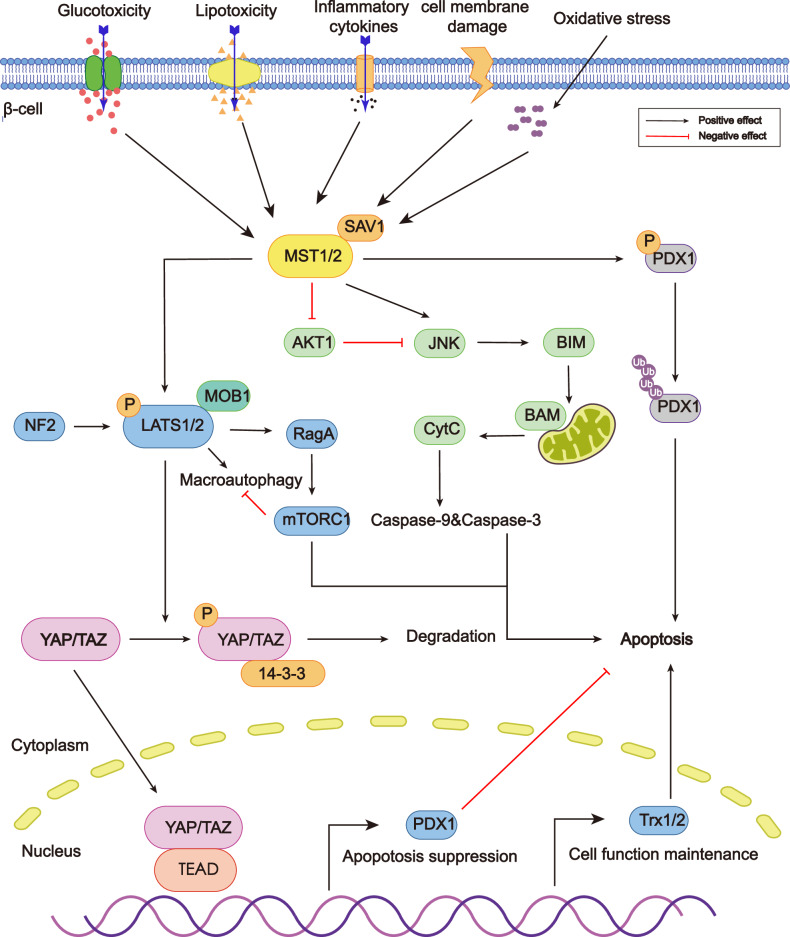
A schematic representation of the crucial function of Hippo signaling pathway in diabetes. External stimuli activate MST1/2 in β-cell and trigger the phosphorylation of downstream effectors, such as PDX1, LATS1/2 and YAP/TAZ. These phosphorylated kinases further lead to diabetes by either interacting with other signaling pathways (e.g., AKT-JNK pathway, mTOR pathway) to promote β-cell apoptosis or inhibiting the normal function of PDX1, which is responsible for insulin production.

As the master contributor of diabetes, β-cell apoptosis can be induced by various external stress factors, such as hyperglycemia, dyslipidemia, glucotoxicity and lipotoxicity, cell membrane damage, oxidative stress, and ER stress [106, 107] (Fig. 5). Therefore, cells are often exposed to such stress conditions to simulate a diabetes condition in relevant studies. β-cell apoptosis can be achieved via two main pathways. The first pathway is activated by the interaction of external molecules and death receptors in the cell membrane, which triggers the activation of downstream caspases. Alternatively, a mitochondrial-dependent pathway is mediated by Bcl-2 family proteins, which orchestrate cell apoptosis by influencing mitochondrial membrane permeability (Fig. 5).

Core kinases in the Hippo signaling pathway are implicated in diabetes progression via modulation of β-cell survival. It regulates the fate of β-cells by facilitating β-cell death directly or suppressing β-cell function, which precedes cell death [108]. Some Hippo kinases, such as MST, have the dual function of promoting β-cell death and loss of function, while others have been reported to work only in one aspect (Fig. 5).

As for the function of β-cell, transcription factors including PDX1 and PAX6 play central roles in maintaining insulin secretion function [109]. Thus, Hippo kinases mainly interact with PDX1 to achieve the dysfunction of β-cells. Ardestani et al. reported that MST1 directly phosphorylated PDX1 at T11, which promoted the ubiquitination and proteasomal degradation of PDX1 in the cytosol [110]. Consequently, the destabilization of PDX1 contributed to the impairment of β-cell function (Fig. 5). Contrary to MST1, downstream Hippo kinases TAZ, act oppositely. Jeong et al. reported that PDX1 binds directly to the N-terminal segment of TAZ. Non-β-cells were able to produce insulin when TAZ was overexpressed ectopically in conjunction with PDX1 activation. TAZ recovery restored normal IPCs differentiation in TAZ-deficient mesenchymal stem cells [111]. Altogether, the over-expression of MST1 and the deficiency of TAZ and Tead1 may interfere with the transcription of PDX1, which further leads to the dysfunction of β-cells and diabetes.

Hippo kinases, including NF2, MST1, LATS1/2, and YAP, have been reported to participate in β-cell death in distinct ways (Fig. 5). In diabetes milieu, MST1 is activated to promote β-cell death via the mitochondrial apoptosis pathway. Meanwhile, Loss of MST1 in mice promoted insulin sensitivity while safeguarding β-cell quantity and performance in the pancreas [110]. As a branch pathway of PI3K-AKT1-JNK signaling pathway, MST1 inhibits AKT1, so that JNK, which is inhibited by AKT1, revives and facilitates β-cell apoptosis [108]. The BCL-2 homology-3 (BH3)-only protein BIM is upregulated by JNK signaling, which then triggers downstream activities of the mitochondria-dependent apoptosis, including the activation of caspase-9 and the cleavage of caspase-3 [110]. In contrast with MST1, YAP has an antiapoptotic effect in β-cells. YAP overexpression is associated with lower expression of cleaved caspase-3 and PARP, and the suppression of apoptosis is achieved via the upregulation of redox protein Trx1/2 [112]. Besides, LATS2 hampers β-cell survival through the autophagy pathway, which further leads to apoptosis. LATS2-MOB1 axis activation is observed under diabetogenic conditions. To further delineate the mechanism, Yuan et al. suggested that LATS2 activated mTORC1 signaling through the RagA GTPase, and subsequently, mTORC1 inhibited macroautophagy, which eventually induced apoptosis [113]. Additionally, under normal conditions, LATS2 can be degraded through the macroautophagy process. However, under prolonged diabetogenic stress, the activation of mTORC1 signaling prevents the degradation of LATS2, leading to its accumulation and the establishment of a negative feedback loop. Yuan et al. also discovered in another study that although NF2 phosphorylated MST in the canonical Hippo pathway, it regulated LATS2 directly in pancreatic β-cells [114], which provided new thoughts for diabetes therapies. These results indicate that overexpression of MST1 and LATS2 and, inversely, inhibition of YAP, induces β-cell apoptosis in diabetes.

### Therapeutic targeting of Hippo signaling pathway in diabetes

Current therapies for diabetes mainly focus on the alleviation of the symptoms without targeting the pathogenesis. Considering the essential role of Hippo kinases in diabetes, potential therapies can be based on Hippo pathway to restore β-cell survival and function. On one hand, the restoration of β-cells can be accomplished by enhancing β-cell self-replication. On the other hand, it can be implemented by targeting core Hippo kinases to attenuate β-cell death.

#### Boosting the natural proliferation of β-cell

The regeneration of β-cells can be accomplished in mainly two aspects: conversion of other cell types into β-cells (neogenesis) [115] and self-replication of β-cells. Neogenesis refers to the process of non-β-cells (α/δ-cells and exocrine cells) getting reprogrammed into β-cells. It has been reported that overexpression of NEUROG3, PDX1 and MAFA can trans-differentiate exocrine cells (especially acinar cells) into β-like cells, while the absence of any one of these transcription factors will lead to the conversion from acinar cells into α/δ-like cells [116]. However, the molecular mechanism of neogenesis is still unclear, and it remains to be explored whether neogenesis has further relation with Hippo signaling pathway.

Hippo pathway has been reported to function in β-cell self-replication. Because YAP is quiescent in β-cells, these cells may regain their proliferative ability with YAP re-expression. Both George et al. and Yuan et al. utilized adenovirus to induce YAP^S127A^ expression in β-cells and thus reported that YAP reintroduction promoted β-cell proliferation efficiently without altering β-cell identity and function [15, 112]. Nonetheless, these two studies provided distinct molecular mechanisms for the same phenotype. George et al. proposed that YAP, through TEAD, activates CTGF and cell cycle components for islet proliferation, or that YAPS127A re-expression activates Akt and mTOR for β-cell replication. Yuan et al. proposed that the robust β-cell proliferation was due to the elevated expression of FOXM1, a downstream effector of YAP [112]. Except for YAP, TEAD1 also plays a critical role in β-cell proliferation. In contrast with YAP, TEAD1 is expressed in β-cells and maintains their proliferative quiescence by activating the transcription of p16^INK4a^. Li et al. demonstrated that VGLL4 and MENIN act as Tead1 corepressors modulating β-cell proliferation, suggesting their potential as targets to promote β-cell regeneration in diabetes [36]. Hence, the deletion of Tead1 in β-cells increases β-cell proliferation.

#### Attenuating β-cell death by targeting core Hippo kinases

MST1 deletion has been proved to improve β-cell survival and insulin secretion in models of diabetes induced by streptozotocin (STZ) or HFD, and a combination of HFD and streptozotocin (HFS/STZ) [105, 112, 113]. Therefore, MST inhibitors may have anti-diabetic functions.

Neratinib, which inhibits MST1, can restore PDX1, Nkx6.1 and GLUT2 expression and help β-cells to regain their function. In both type 1 (STZ-induced) and type 2 (obese Lepr^db/db^) diabetic mouse models, decreased β-cell apoptosis and increased cell mass can be observed after neratinib treatment [117, 118]. However, it may have gastric and intestinal side effects because it inhibits EGFR. XMU-MP-1 can also inhibit MST1 and restore β-cell in ex vivo INS-1 cells and STZ-induced diabetic mice. Additionally, IHMT-MST1-39, a MST1 inhibitor, has demonstrated significant anti-apoptotic effects, effectively enhancing the survival of pancreatic β-cells under diabetes-inducing conditions and improving the viability of primary islets in ex vivo disease models [119].

Similarly, LATS2 ablation achieved by siRNA in β-cells can protect from both STZ and HFD-induced diabetes [113]. Highly associated with LATS2, inhibition of NF2 by siRNA can efficiently block LATS2 and protect β-cells from apoptosis [114]. Nonetheless, due to the unknown crystal structures of the LATS kinases, few LATS2 inhibitors have been found. Recent studies revealed that TRULI might inhibit LATS2 efficiently, but at the risk of disrupting other enzymes [120]. Therefore, more inhibitors of NF2 and LATS2 are expected to be designed and tested in the pancreatic β-cells.

Contrary to the absence of MST1/2 or LATS1/2, the activation of YAP/TAZ in β-cells has been confirmed important to β-cell survival and function in ex vivo experiments [111, 112]. It implies that maintaining the expression of YAP/TAZ, which can be implemented by YAP/TAZ/TEAD1 activators or inhibitors of upstream kinases (MST1/2, LATS1/2), maybe a potential target in future diabetes treatment.

## Conclusions and further perspectives

Dysregulation of Hippo pathway activity is closely associated with the development and progression of various pancreatic diseases. For instance, upregulation of YAP and suppression of upstream Hippo kinases are considered key prognostic factors in PDAC. Elevated YAP expression promotes ADM, proliferation of pancreatic progenitor cells, EMT, and activation of tumor microenvironment-associated signaling pathways. Similarly, YAP activation is also observed in pancreatitis, which is recognized as an important early risk factor for PDAC.

In contrast to PDAC and pancreatitis, diabetes mellitus is characterized by activation of upstream Hippo regulators and functional suppression of YAP and TEADs. This dysregulation induces apoptosis of pancreatic β-cells through crosstalk with other signaling pathways, including mTORC1 and AKT, thereby exacerbating disease progression.

Although recent studies have begun to elucidate the role of YAP in pancreatic pathologies, it remains unclear whether aberrant YAP expression is driven by upstream Hippo signaling components. The specific regulatory roles of other Hippo kinases in modulating YAP activity also warrant further investigation. Given YAP’s crucial physiological functions in pancreatic development, regeneration, and disease, future therapeutic strategies should focus on restoring normal YAP expression in pancreatic tissues. For example, the development of pharmacological agents or gene therapies that inhibit YAP nuclear translocation or disrupt YAP–TEAD interactions may offer effective approaches to suppress the progression of PDAC and pancreatitis.
